# The bioinformatics analysis and experimental validation of the carcinogenic role of EXO1 in lung adenocarcinoma

**DOI:** 10.3389/fonc.2024.1492725

**Published:** 2024-12-24

**Authors:** Bohao Sun, Jing Zhang, Nan Wang, Zhirong Zhang, Yichen Wu, Mengzhen Xie, Yanmei Peng, Yifan Ye, Zhaochang Jiang, Shumei Wei

**Affiliations:** ^1^ Department of Pathology, Second Affiliated Hospital, School of Medicine, Zhejiang University, Hangzhou, Zhejiang, China; ^2^ School of Pharmaceutical Science, Wenzhou Medical University, Wenzhou, Zhejiang, China; ^3^ Faculty of Medicine and Life Sciences, Xiamen University, Xiamen, China; ^4^ Zhejiang Provincial Key Laboratory of Medical Genetics, School of Laboratory Medicine and Life Sciences, Wenzhou Medical University, Wenzhou, Zhejiang, China

**Keywords:** LUAD, EXO1, poor-prognosis predictor, clinical stage, bioinformatics analysis

## Abstract

**Background:**

Exonuclease 1 (EXO1), a protein involved in mismatch repair and recombination processes, has been identified as a prognostic biomarker in lung adenocarcinoma (LUAD). Nevertheless, its role in LUAD progression remains elusive. This study seeks to elucidate the functional significance of EXO1 in LUAD and evaluate its potential as a therapeutic target.

**Materials and methods:**

Patient RNA-seq and clinical data were acquired from The Cancer Genome Atlas (TCGA) and Gene Expression Omnibus (GEO) databases. Subsequently, a protein-protein interaction (PPI) network was constructed using differentially expressed genes (DEGs) to identify pivotal genes. Validation of the expression of signature genes was carried out through quantitative real-time PCR (qRT-PCR). Additionally, the association between EXO1 expression and clinical data was investigated. Immunohistochemistry was utilized to assess EXO1 expression in 93 cases of invasive pulmonary adenocarcinoma. Finally, cellular functional assays were conducted to investigate the impact of EXO1 on LUAD cells.

**Results:**

Ten key molecules (PBK, ASPM, NCAPG, EXO1, MKI67, RRM2, AURKA, DLGAP5, UBE2C, and CDC6) exhibited significantly elevated expression levels in LUAD tissues. Moreover, elevated levels of EXO1 gene expression correlated strongly with advanced T, N, and M stages and were significantly associated with immune cell infiltration in LUAD. Furthermore, marked increases in EXO1 protein expression were observed in patients diagnosed with invasive pulmonary adenocarcinoma. Notably, patients diagnosed with invasive pulmonary adenocarcinoma who exhibited elevated EXO1 expression levels exhibited increased lymph node metastasis, pleural invasion, poor tumor differentiation, and advanced clinical stage. Additionally, this study employed wound healing assay and CCK-8 cell proliferation assays to investigate the significant role of EXO1 in promoting the growth and migration of lung adenocarcinoma cells.

**Conclusions:**

This study identified ten hub genes associated with the initiation and progression of LUAD. Additionally, EXO1 may serve as a prognostic marker for LUAD patients, offering new perspectives for clinical treatments.

## Introduction

Lung cancer is among the most prevalent malignancies globally, with annual increases in incidence and mortality rates in both genders. This exerts a substantial burden on families and society (1, 2). Histologically, small cell lung cancer (SCLC) constitutes 15% of cases, while the remaining 85% fall into the non-small-cell lung cancer (NSCLC) category (3, 4). Among NSCLC cases, lung adenocarcinoma (LUAD) stands as the predominant subtype. Despite significant advances in understanding the mechanisms and technological improvements that have reshaped early-stage LUAD treatment strategies over the past decade, a considerable portion of advanced LUAD patients lack actionable mutations, resulting in a 5-year survival rate of less than 15% (5, 6).

Due to the lack of effective strategies for early diagnosis and treatment of LUAD, the postoperative survival rate for patients remains suboptimal. Patient outcomes following surgery are significantly influenced by tumor metastasis and recurrence. As a result, we are dedicated to developing multiple immune biomarkers and gene signatures associated with immune infiltration, with the potential to predict responses to immunotherapy in LUAD cases. Over the past few decades, extensive genomic studies have revealed many common genetic alterations in LUAD, including EGFR and KRAS mutations, as well as ALK rearrangements. These genetic variations are likely involved in the initiation and progression of LUAD, leading to extensive drug development efforts centered around EGFR signaling pathways (7–9).

In recent years, the advancement of next-generation high-throughput sequencing technologies has led to the accumulation of extensive genetic data and clinical information within various public databases, including the Cancer Genome Atlas Database (TCGA) (10, 11). This sets the groundwork for deepening the understanding of the molecular mechanisms of cancer and the biological roles of key genes through bioinformatics techniques and experimental validation.

EXO1, short for Exonuclease 1, belongs to the Aspartyl-Cysteinyl-Asparagine (APC) enzyme family (12). Members of this family play crucial roles in various cellular processes, including cell cycle regulation, DNA damage repair, cell proliferation, and apoptosis (13–15). Aberrant activation of APC enzymes may contribute to tumorigenesis by promoting the growth and spread of cancer cells (16). Expression levels of EXO1 vary across different cancers; for instance, it is elevated in certain tumors such as lung adenocarcinoma (17), breast cancer (18), and hepatocellular carcinoma (19), while reduced in others like neuroblastoma (20) and leukemia (21). This differential expression suggests the potential of EXO1 as a drug target and provides insights into the biological mechanisms within the tumor microenvironment. However, the relationship between EXO1 expression and clinical factors as well as prognosis in lung cancer remains unexplored, necessitating further research to elucidate its role in cancer progression. Our comprehensive study identifies potential genetic biomarkers, offering new insights into therapeutic targets for LUAD.

## Materials and methods

### Gene expression profiles of LUAD and adjacent normal lung tissues

Gene expression data, clinical survival information, and gene mutation data for patients diagnosed with LUAD were acquired from TCGA database (TCGA-LUAD) and the Gene Expression Omnibus (GEO) database (accession numbers: GSE31210, GSE115002). Specifically, the dataset included 526 LUAD tissue samples and 59 normal lung tissue samples from the TCGA database, 226 LUAD tissue samples and 20 normal lung tissue samples from GSE31210, and 52 LUAD tissue samples and 52 normal lung tissue samples from GSE115002.

### Identification and analysis of differentially expressed genes

Normalization of gene expression data and identification of differentially expressed genes were performed using the R limma package (Version 3.18) (22). The criteria for selecting significant DEGs were set at a p-value of < 0.05 and a logFC of > |1|.

### Multi-omics analyses of identified genes in LUAD

We constructed a PPI network of proteins expressed by DEGs using the STRING database (https://string-db.org/). To identify the most significant modules within the PPI network, we employed the Cytoscape plugin molecular complex detection (MCODE), which is based on graph-theoretic clustering algorithms (23). Selection criteria were established as K-core = 2, degree cutoff = 10, max depth = 100, and node score cutoff = 0.2. Key genes within the PPI network, showing high connectivity and substantial prognostic significance, were determined (24). Mutation data of LUAD patients were obtained from the TCGA database. The Human Protein Atlas (HPA) database (https://www.proteinatlas.org/) was used to validate the protein expression of these key genes in LUAD and normal tissues (25). The images were available from version 23.0.

### Construction of prognostic signatures based on key genes

Building on the collective impact of these ten genes in LUAD progression, we developed risk score signatures to comprehensively examine their role in patient prognosis, immune cell infiltration, and immunotherapeutic response. The risk score signature was formulated using the most robust markers, identified through LASSO Cox regression (26). The optimal penalty parameter lambda was determined through 10-fold cross-validation.

### Patients and tissue samples

The LUAD tissues and the adjacent paracancerous tissues were obtained from the Department of Pathology, Second Affiliated Hospital, School of Medicine, Zhejiang University. The present study adhered to the ethical principles outlined in The Declaration of Helsinki. The study’s inclusion criteria consisted of the following: i) presence of a primary lung adenocarcinoma; ii) histopathological verification of lung adenocarcinoma diagnosis; and iii) availability of complete clinical records. Conversely, the exclusion criteria comprised: i) presence of autoimmune disorders or other diseases; ii) presence of other severe illnesses; and iii) history of prior immunosuppressive treatments. Study procedures were approved by the Ethics Committee of the Second Affiliated Hospital of Zhejiang University School of Medicine, Hangzhou, China (approval no: 2023-0970). Patient follow-up started in June 2020 and continued until November 2023. Since the present study was retrospective and non-interventional in nature, the requirement to obtain patient informed consent for participation was waived by the Ethics Committee. A total of 93 patients diagnosed with invasive lung adenocarcinoma were selected for this study. These patients had previously undergone surgical resection at the Second Affiliated Hospital of Zhejiang University School of Medicine. The samples of LUAD and their corresponding medical information were collected.

### Quantitative real-time PCR

Total RNA was extracted from lung adenocarcinoma tissues of patients following the manufacturer’s instructions using a kit from XiaMen AmoyDx. Reverse transcription was carried out with PrimeScript RT Master Mix at 37°C for 15 minutes followed by 85°C for 5 seconds (Takara). Takara Biotechnology TB Green Premix Ex Taq™ II (cat. no. RR820A) was employed for qPCR, with the following thermocycling conditions: initial denaturation at 95°C for 30 seconds, followed by 40 cycles of 95°C for 5 seconds and 60°C for 30 seconds, and a final extension at 95°C for 15 seconds and 60°C for 60 seconds. The relative expression levels of ten genes were quantified employing the 2−ΔΔCq method (27), with β-actin serving as an internal control (Takara). The primers were designed by Qingke Biotechnology Co. The primers used in this study are shown in the **Supplementary Table S1**.

### Gene set enrichment analysis

GSEA was performed using the clusterProfiler package in R, where gene sets with normalized enrichment scores (NES) exceeding 1 and false discovery rates (FDR) below 0.05 were considered significant.

### Immunoinfiltration analysis

We conducted an analysis of immune cell infiltration using the ssGSEA algorithm (28). We compared distinct gene sets characteristic of immune cells and transformed sample gene expression values into enrichment fractions. This approach enabled us to derive the relative abundance of immune cells using the GSVA R package (Version 1.34).

### Western blot analysis

γH2AX expression level in A549 cell were evaluated using Western blot analysis. Initially, cells were lysed with lysis buffer, and protein concentrations in the lysates were measured by the BCA assay (Beyotime Institute of Biotechnology, Shanghai, China). Subsequently, 10 µg of each lysate was separated on 10% SDS-PAGE and transferred to PVDF membranes (EMD Millipore). To reduce non-specific binding, membranes were blocked using 5% non-fat dry milk in TBST. Immunoblotting was performed using an anti-γH2AX antibody (1:1,000; HUABIO), an anti-EXO1 antibody (1:1,000; HUABIO), and an HRP-conjugated anti-rabbit secondary antibody (1:5,000; Santa Cruz Biotechnology, Inc., CA, USA). Blots were developed with ECL reagent (Beyotime Institute of Biotechnology) and imaged with the ChemiDoc MP Imaging System (Bio-Rad Laboratories, Inc.).

### Immunohistochemistry

Tissue sections (2.5µm) mounted on glass microscope slides were deparaffinized in xylene and rehydrated in graded alcohols. Then the tissue sections were boiled for 15 min in 10 mM citric acid buffer (pH 6.0) for antigen retrieval. After the sections were cooled to room temperature (RT), they were incubated with 3% H_2_O_2_ for 15 min at 37°C to block endogenous peroxidase activity. Then the sections were blocked with 5% goat serum (Scientific Phygene) for 45 min at room temperature, and incubated with primary antibodies, including EXO1 (1:300; HuaBio), MKI67 (1:1,000; Zhongshan Golden Bridge Biotechnology Co), TTF1 (1:1,000; Zhongshan Golden Bridge Biotechnology Co), and NapsinA (1:1,000; Zhongshan Golden Bridge Biotechnology Co), overnight at 4°C. Subsequently, the sections were incubated with secondary antibodies, including goat anti-rabbit IgG antibody (1:1,000; Abcam), for 30 min. Finally, the substrate color was developed using a diaminobenzidine substrate kit (Abcam), and the sections were counterstained with hematoxylin for 30s. Image acquisition was performed using a light microscope (Nikon Corporation). Immunohistochemistry results were quantified using ImageJ software (version 1.8.0).

### Cell lines

The A549 and H322 cell lines were sourced from the American Type Culture Collection (ATCC) in Maryland, USA. A549 and H322 cells were cultured in DMEM/F12 medium (Gibco, California, USA) supplemented with 10% fetal bovine serum (Lonsera, Australia) and 1% penicillin/streptomycin (Beyotime Biotechnology, Jiangsu, China).

### Plasmids

EXO1-shRNA1 and shRNA2 were subcloned into EcoRI and AgeI digested pLKO.1vector. All generated plasmids were verified by DNA sequencing (Qingke Biotechnology Co.).

### Lentivirus production and viral infection

The production of lentiviruses and viral infections of cells were performed previously as described (29). To generate recombinant lentivirus, 293T-17 cells were co-transfected with three plasmids—pLEX-CMV-EXO1, pSPAX2, and pMD2G (SBI System Biosciences, Palo Alto, CA, USA)—using polyethyleneimine (PEI) at a 4:3:1 ratio. Lentiviral supernatants were collected 48 and 72 hours post-transfection, centrifuged at 3,000 rpm for 10 minutes, and filtered through a 0.45 µm membrane before storage at -80°C. Target cells were then infected with EXO1-recombinant lentivirus in the presence of 8 µg/ml polybrene and subsequently selected with 2.5 µg/ml puromycin (Invitrogen, San Diego, USA) for four days.

### Wound healing assay

For the wound-healing assay, cells were seeded in 24-well plates. Cells were scratched with a sterile tip perpendicular to the previously painted line. Scratch wounds were imaged using a light microscope after which cell migration was measured at indicated time points of 0 and 48 h. All experiments were repeated three times.

### Cell proliferation assays

Cells were collected by centrifugation and resuspended in the medium at a density of 10^4^ cells per milliliter of medium. Then, 200 μl of culture medium was added to each well of a 96-well culture plate. Cell proliferation assays were conducted using CCK-8 (Beyotime Biotechnology, Shanghai) and measured at 0, 24, 48, and 72 hours following the manufacturer’s instructions. The optical density of the cells at 450 nm (OD450) was measured and recorded using GraphPad Prism software to assess their proliferative ability. All experiments were repeated three times.

### Statistical analysis

Statistical and bioinformatics analyses were conducted using R software (version 4.2.0). One-way analysis of variance (ANOVA) was used to compare multiple groups, followed by Tukey’s *post hoc* test for pairwise comparisons. For comparisons between the two groups, we employed the Wilcoxon rank-sum test, while correlation analyses were based on Spearman and distance correlation methods. The Kaplan-Meier method was used for the survival curve data. The survival period was defined as the time from the start of treatment to the end of the observation.

## Results

### The genomic differences between normal lung and LUAD tissues

**Figure 1:**
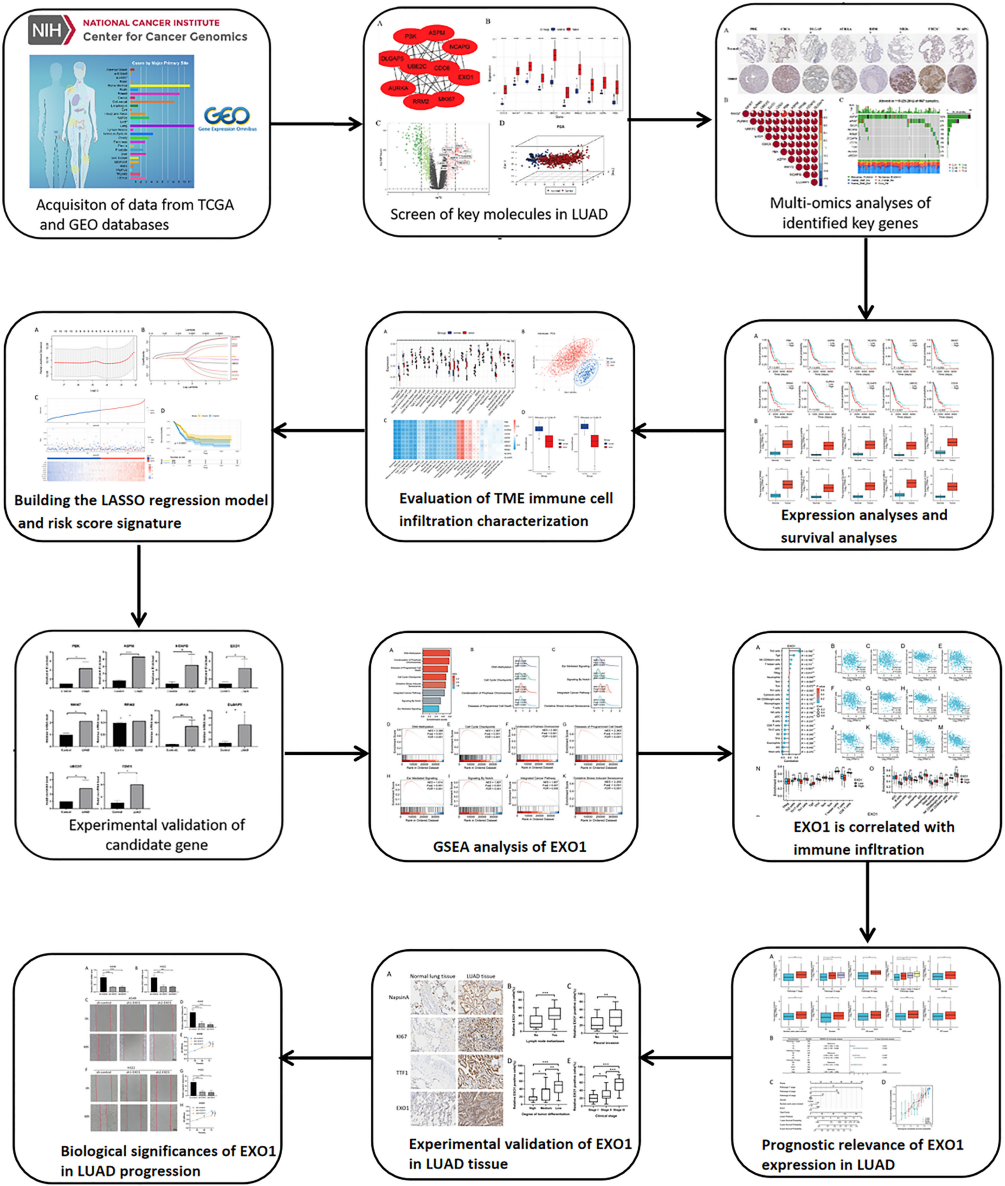
The workflow of the research.

**Figure 2:**
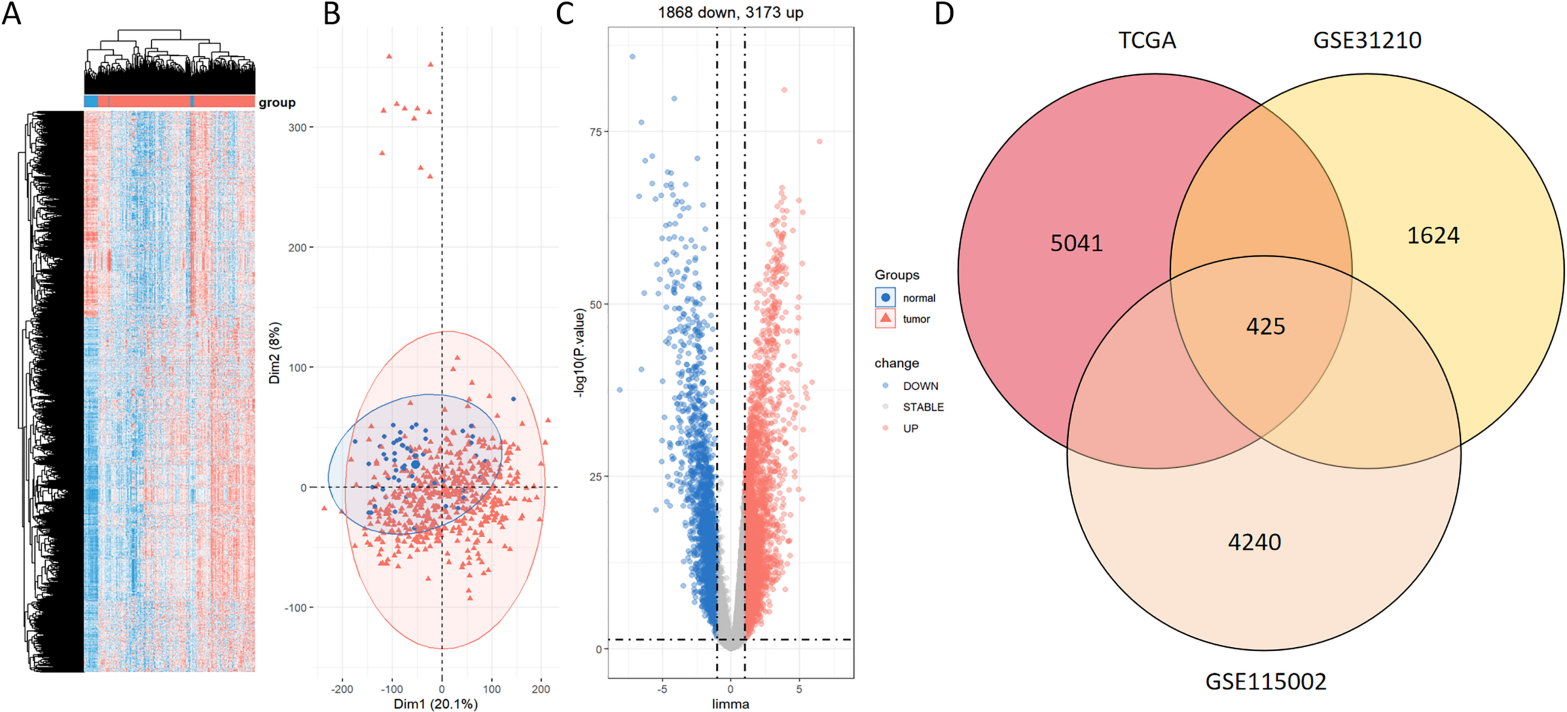
Identification of DEGs between normal and LUAD groups within the TCGA dataset. **(A)** Heatmap displaying prominently upregulated (red) and downregulated (blue) genes in tumor and normal groups under the threshold of log2 (fold change) > 1 and p < 0.05. **(B)** PCA analysis contrasting tumor (red) and normal (blue) groups. **(C)** Volcano plot portraying conspicuously upregulated (red) and downregulated (blue) genes in tumor and normal groups under the threshold of log2 (fold change) > 1 and p < 0.05. **(D)** Venn diagram of the DEGs between TCGA, GSE31210, and GSE115002.

**Figure 1** presents a flow diagram elucidating the study’s procedural framework. In a comprehensive scope, our investigation involved 59 normal samples and 526 tumor samples from TCGA, 20 normal samples and 226 tumor samples from GSE31210, and 52 normal samples and 52 tumor samples from GSE115002. Systematic clustering clearly delineated genomic disparities between normal and LUAD tissues (**Figure 2A**; **Supplementary Figures S1A**, **S2A**). Principal Component Analysis (PCA) underscored a marked separation between the control and tumor groups, with minimal overlap between them (**Figure 2B**; **Supplementary Figures S1B**, **S2B**). The volcano plot of differentially expressed genes highlighted up-regulated and down-regulated genes in LUAD tissues compared to normal tissues (**Figure 2C**; **Supplementary Figures S1C**, **S2C**). The Venn diagram demonstrates the presence of 425 differentially expressed genes across the three datasets (**Figure 2D**).

### Screen of key molecules in LUAD

**Figure 3:**
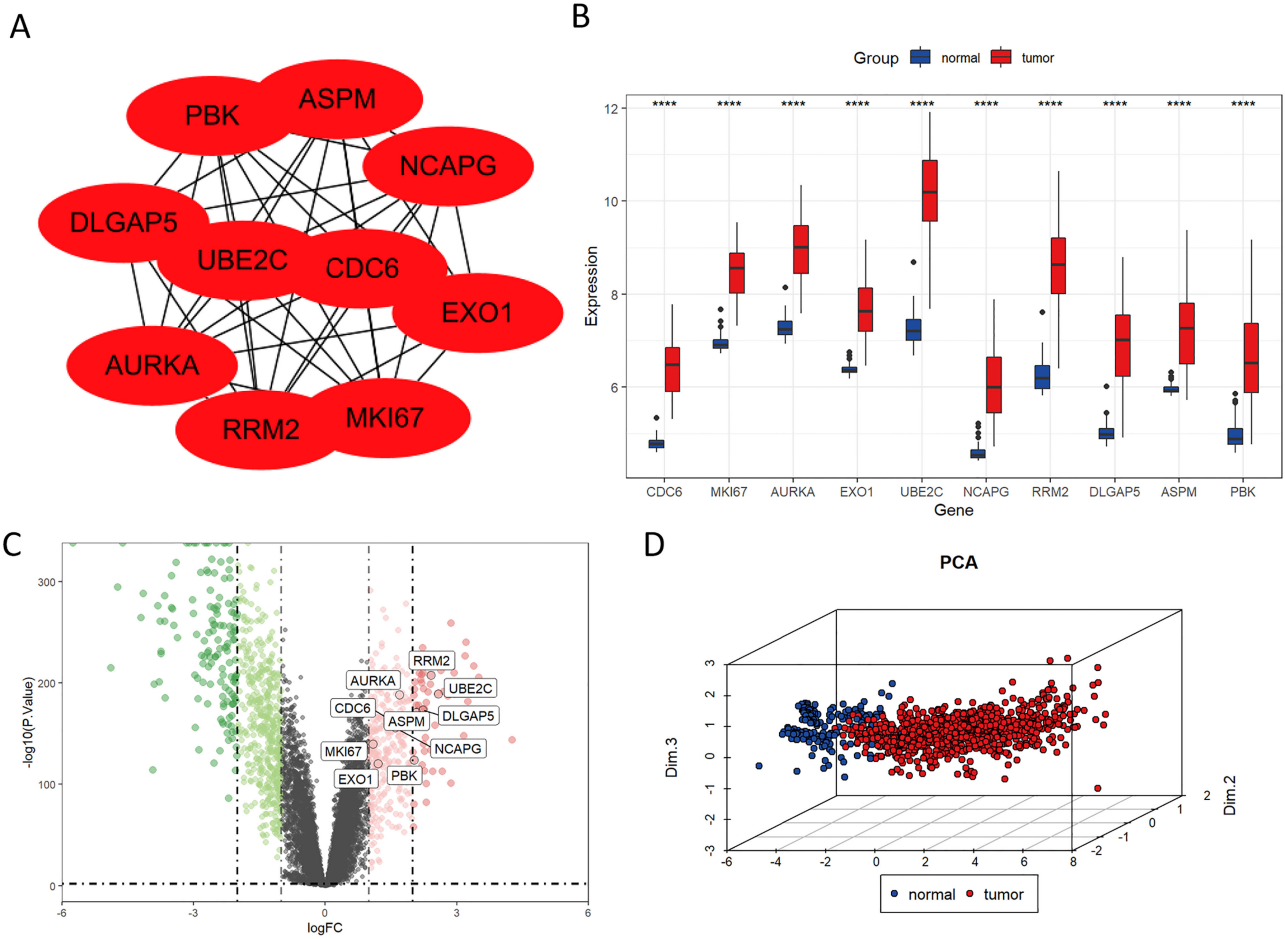
Identification of key molecules in LUAD. **(A)** Protein-level interaction network featuring the 10 key molecules. **(B)** The mRNA expression levels of CDC6, MKI67, AURKA, EXO1, UBE2C, NCAPG, RRM2, DLGAP5, ASPM, and PBK were significantly elevated in LUAD tissues compared to normal tissues in the TCGA database. **(C)** Volcano plot illustrating significantly upregulated (red) and downregulated (blue) genes in tumor and normal groups under the threshold of log2 (fold change) > 1 and p < 0.05. **(D)** Principal Component Analysis (PCA) of key molecules. ****P < 0.0001.

We established a PPI network to investigate the interrelationships among the protein-encoding DEGs associated with LUAD. Among these DEGs, the top ten genes, namely PBK, ASPM, NCAPG, EXO1, MKI67, RRM2, AURKA, DLGAP5, UBE2C, and CDC6, were identified as key molecules due to their pronounced connectivity (**Figure 3A**). It is noteworthy that the expressions of these key molecules were significantly elevated in tumor samples (**Figures 3B, C**). Principal Component Analysis (PCA) revealed two distinct populations, highlighting a significant difference in key molecule expression between normal samples and LUAD tumors (**Figure 3D**).

### Multi-omics analyses of identified key genes

**Figure 4:**
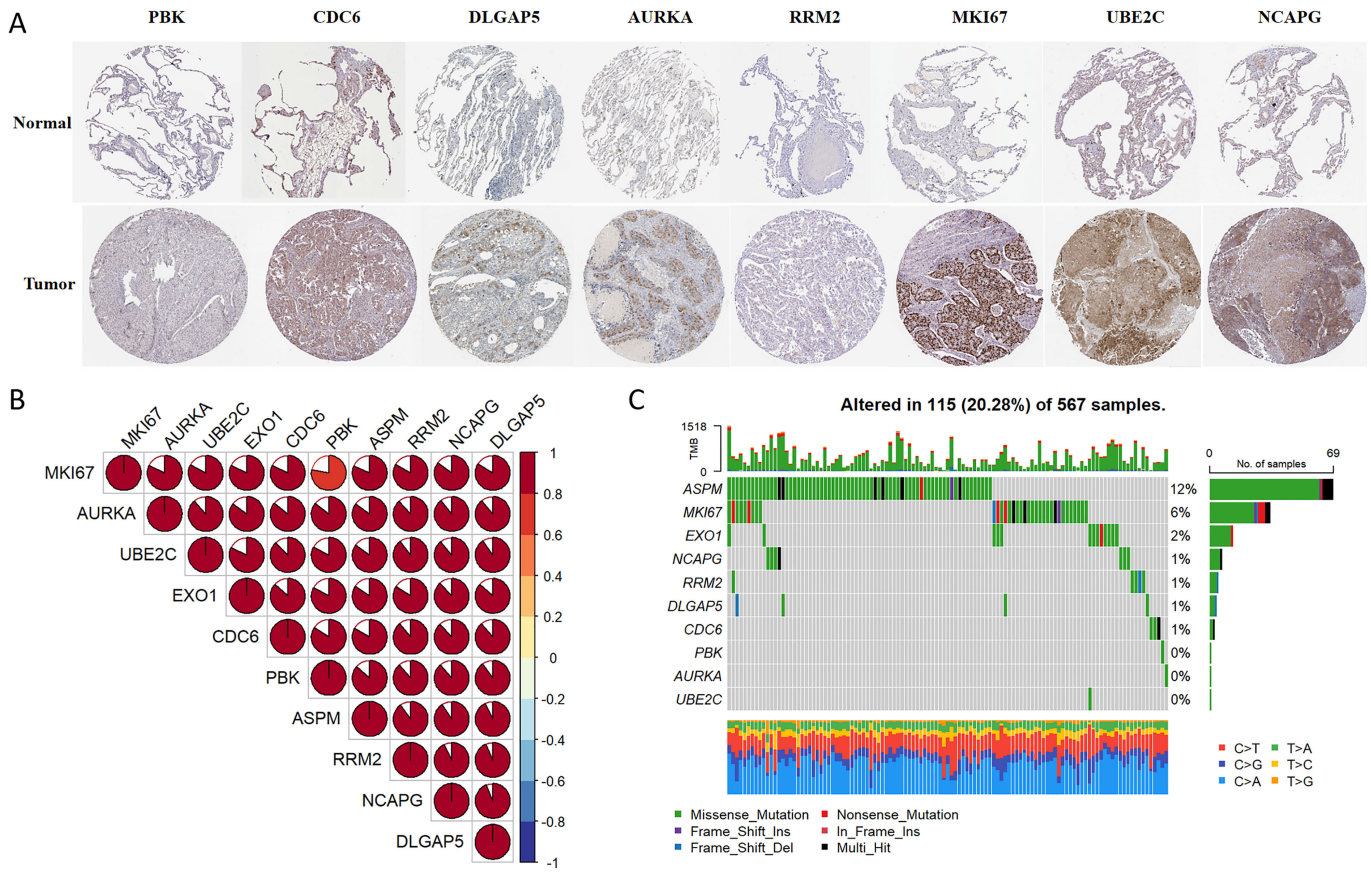
Multi-omics analyses of identified key molecules. **(A)** The protein expression levels of PBK, CDC6, DLGAP5, AURKA, RRM2, MKI67, UBE2C, and NCAPG showed significant elevation in LUAD tissues compared to normal tissues according to the HPA database (https://www.proteinatlas.org/). The images were available from version 23.0. **(B)** Correlation among the 10 key molecules through Spearman analyses. **(C)** Mutation landscape of key molecules within a cohort of 567 LUAD samples.

**Figure 5:**
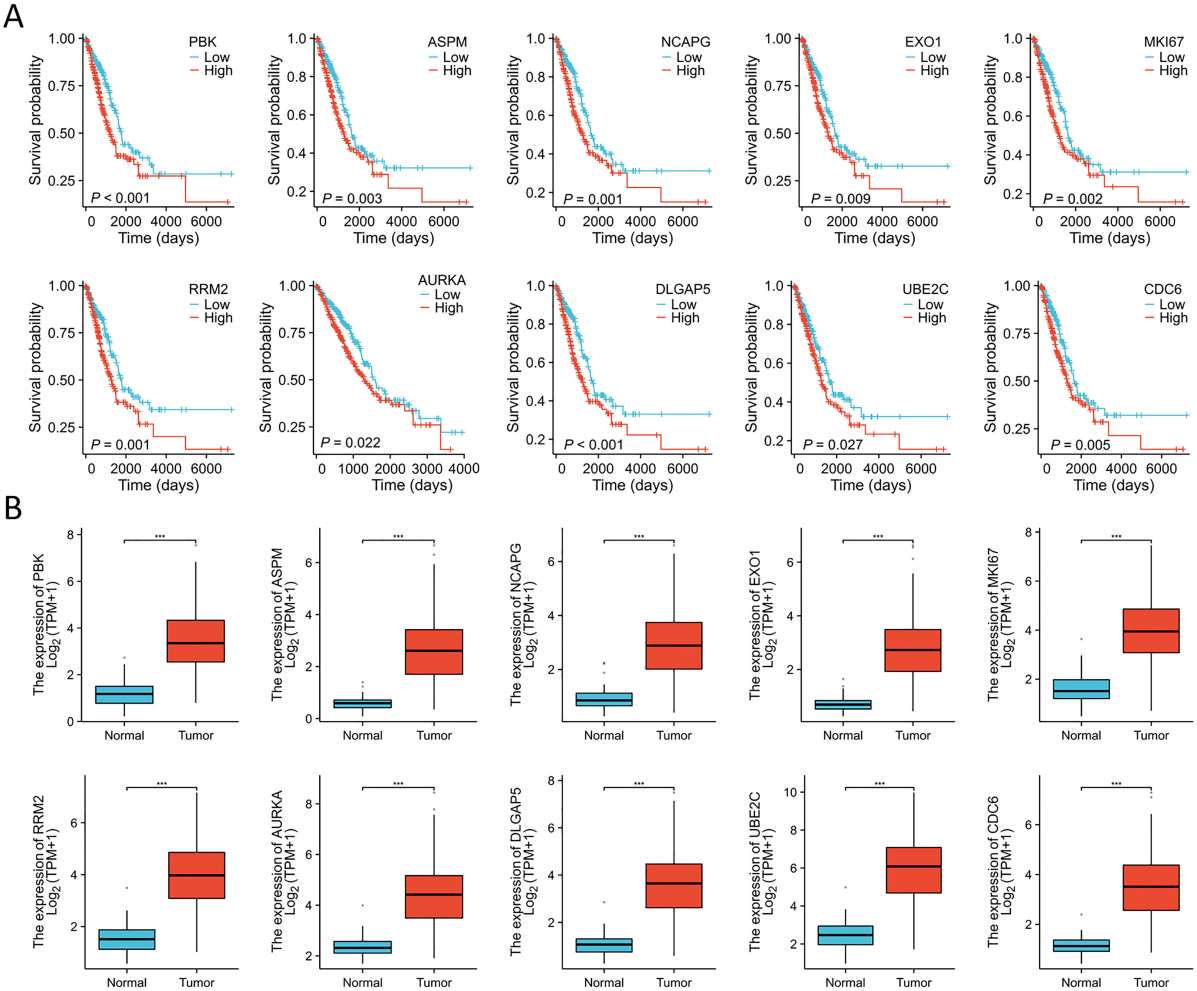
Expression analyses and survival analyses for 10 key molecules. **(A)** Decreased expression of PBK, ASPM, NCAPG, EXO1, MKI67, RRM2, AURKA, DLGAP5, UBE2C, and CDC6 in LUAD is associated with substantial survival benefits according to the TCGA database. **(B)** The expression of PBK, ASPM, NCAPG, EXO1, MKI67, RRM2, AURKA, DLGAP5, UBE2C, and CDC6 were elevated in LUAD tissues compared to the normal tissues in the TCGA database. ***P < 0.001.

Immunohistochemistry staining results for eight key genes were obtained from the Human Protein Atlas (HPA) database. In contrast to the normal group, the protein expression levels of PBK, CDC6, DLGAP5, AURKA, RRM2, MKI67, UBE2C, and NCAPG were substantially increased in the tumor group (**Figure 4A**). We performed a Spearman correlation analysis to examine the mRNA expression levels of 10 key molecules. The analysis identified a significant positive correlation among these molecules in LUAD (**Figure 4B**). Subsequently, we explored the mutational landscape of these genes in LUAD. Out of the 567 samples assessed, approximately 20.28% of patients exhibited mutations in at least one key molecule. ASPM exhibited the highest mutation frequency, closely followed by MKI67 (**Figure 4C**). Furthermore, LUAD patients with lower expressions of PBK, ASPM, NCAPG, EXO1, MKI67, RRM2, AURKA, DLGAP5, UBE2C, and CDC6 enjoyed a significant survival advantage compared to those with elevated expression (**Figure 5A**). These findings underscored the evident disparities in the expression of selected target genes between normal and tumor tissues. Moreover, the expression of these key molecules within LUAD tissues was markedly increased in comparison to normal tissues (**Figure 5B**).

### Construction of prognostic and immunotherapeutic risk model

**Figure 6:**
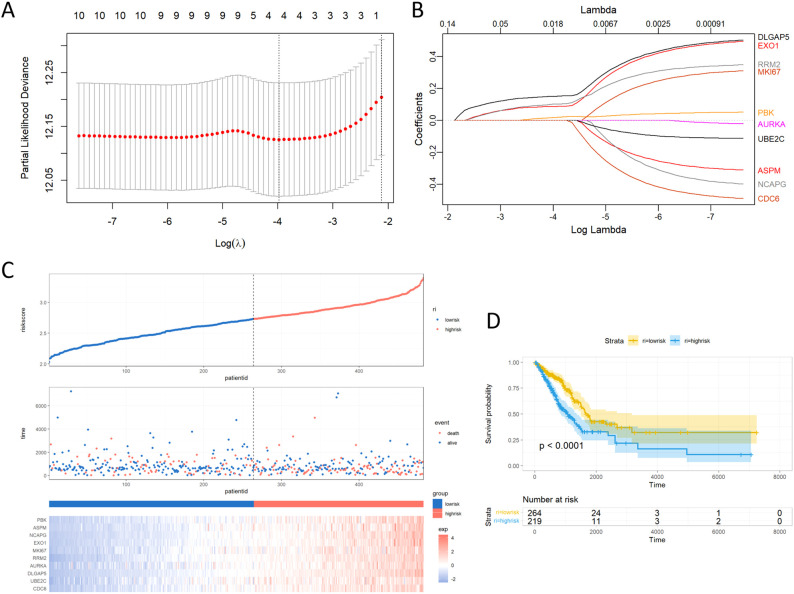
Building the LASSO regression model and risk score signature. **(A)** Tuning parameter selection through tenfold cross-validation in the LASSO model. **(B)** Coefficient profiles of the 10 key genes as derived from the LASSO method. **(C)** Proportion of mortality as risk score values escalated within low and high-risk groups. **(D)** Survival analyses presented through Kaplan-Meier curves, differentiating low and high-risk score groups.

To further explore the clinical significance of the regulatory network orchestrated by the 10 key genes, a risk score signature was expertly fashioned to consolidate the contributions of these 10 pivotal genes, using the LASSO Cox regression model (**Figures 6A, B**). The findings emphasized that the risk score depended on the expression of the four most influential key molecules: DLGAP5, EXO1, RRM2, and PBK. Manifestly, as the risk value increased, a consequential escalation in patient mortality was evident (**Figure 6C**). Revealing the outcome of KM survival analysis, it was distinctly discerned that individuals classified in the low-risk group enjoyed superior survival advantages (**Figure 6D**).

### Experimental verification of the expression of key molecules

**Figure 7:**
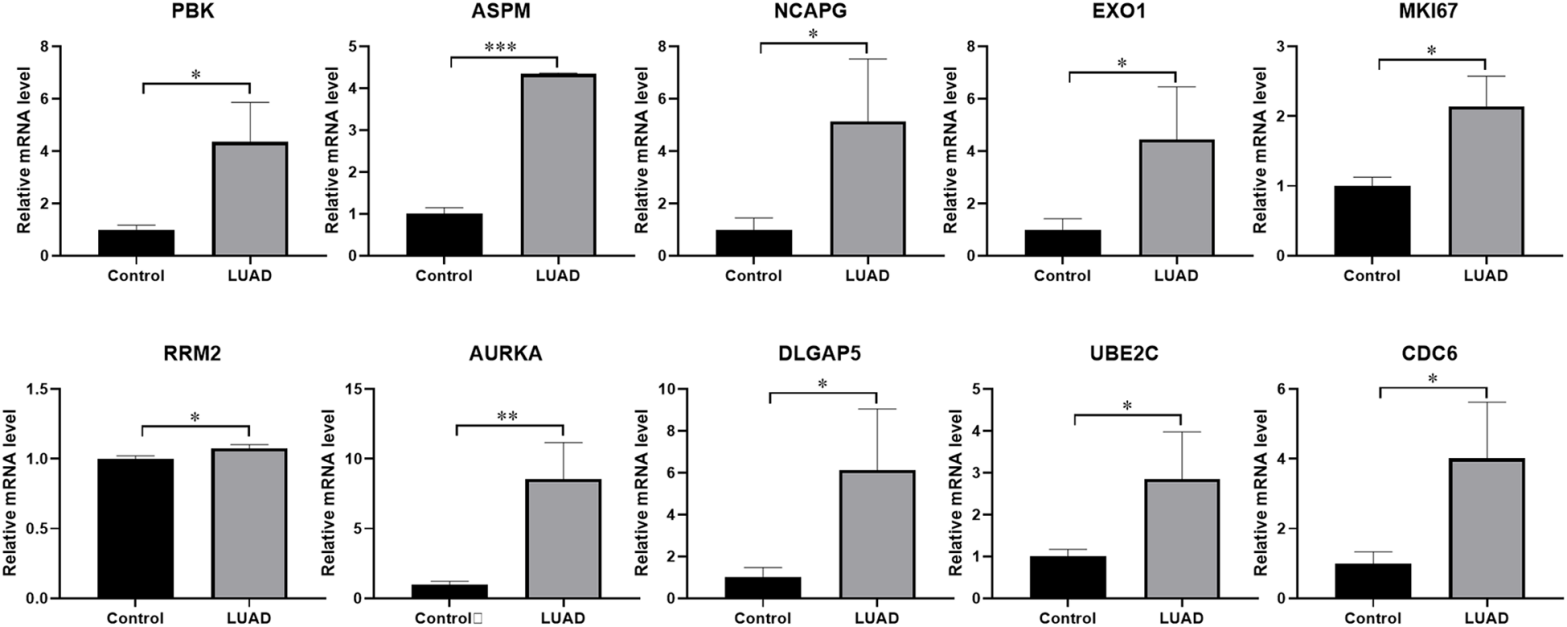
The expression levels of key molecules detected by qRT-PCR. The mRNA expression levels of PBK, ASPM, NCAPG, EXO1, MKI67, RRM2, AURKA, DLGAP5, UBE2C, and CDC6 were notably higher in LUAD tissues than in normal tissues. *P < 0.05, **P < 0.01, ***P < 0.001. Samples (n) = 3.

To investigate the variations in the mRNA expression of key moleculars between normal and LUAD tissues, we examined the expression levels of PBK, ASPM, NCAPG, EXO1, MKI67, RRM2, AURKA, DLGAP5, UBE2C, and CDC6 in tissues by using qRT-PCR. The findings indicate that crucial molecules showed significantly higher expression levels in LUAD tissues compared to the adjacent paracancerous tissues (**Figure 7**).

### GSEA analysis of EXO1 in LUAD

**Figure 8:**
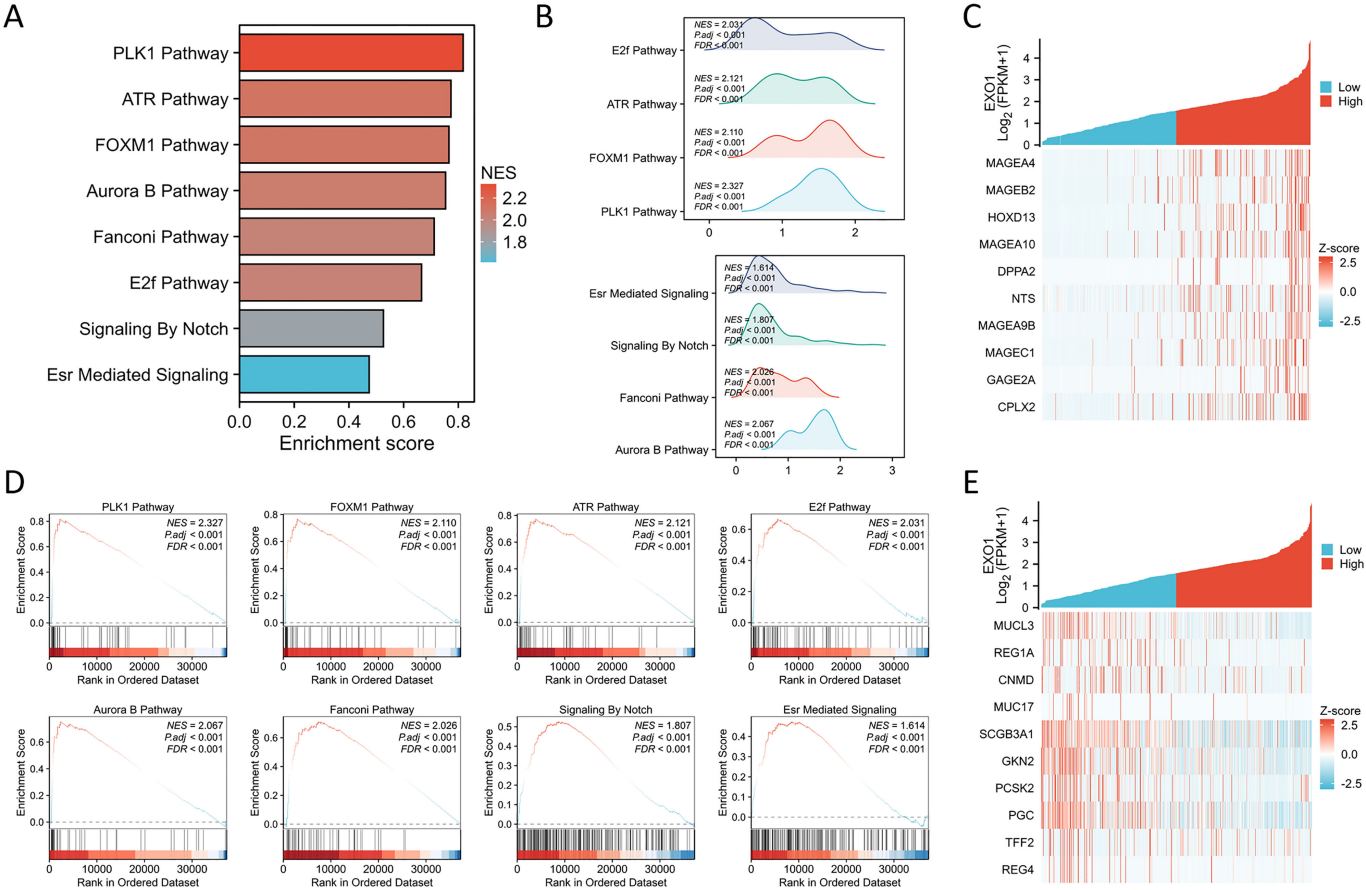
GSEA of EXO1. **(A)** The results of the GSEA enrichment are illustrated in a bar chart format. **(B)** The GSEA enrichment outcomes are represented through mountain plots. **(C)** The top 10 positively co-expressed genes of EXO1 shown in heat maps. **(D)** Analysis of the functional roles and pathway enrichment associated with EXO1. **(E)** The top 10 negatively co-expressed genes of EXO1 shown in heat maps.

GSEA demonstrated that the expression of EXO1 is significantly associated with several key molecular mechanisms, which include the essential pathways of PLK1, FOXM1, ATR, E2F, AURORA_B, and FANCONI, in addition to the critical Notch signaling and ESR-mediated signaling pathways (**Figures 8A, B, D**). These compelling findings reveal the intricate potential molecular mechanisms through which EXO1 may promote both cell growth and migration in lung adenocarcinoma (LUAD), suggesting a multifaceted role in the progression of this disease. In addition, the top 10 genes that were positively and negatively linked with EXO1 were exhibited on heat maps (**Figures 8C, E**).

### EXO1 is correlated with immune infiltration in LUAD

**Figure 9:**
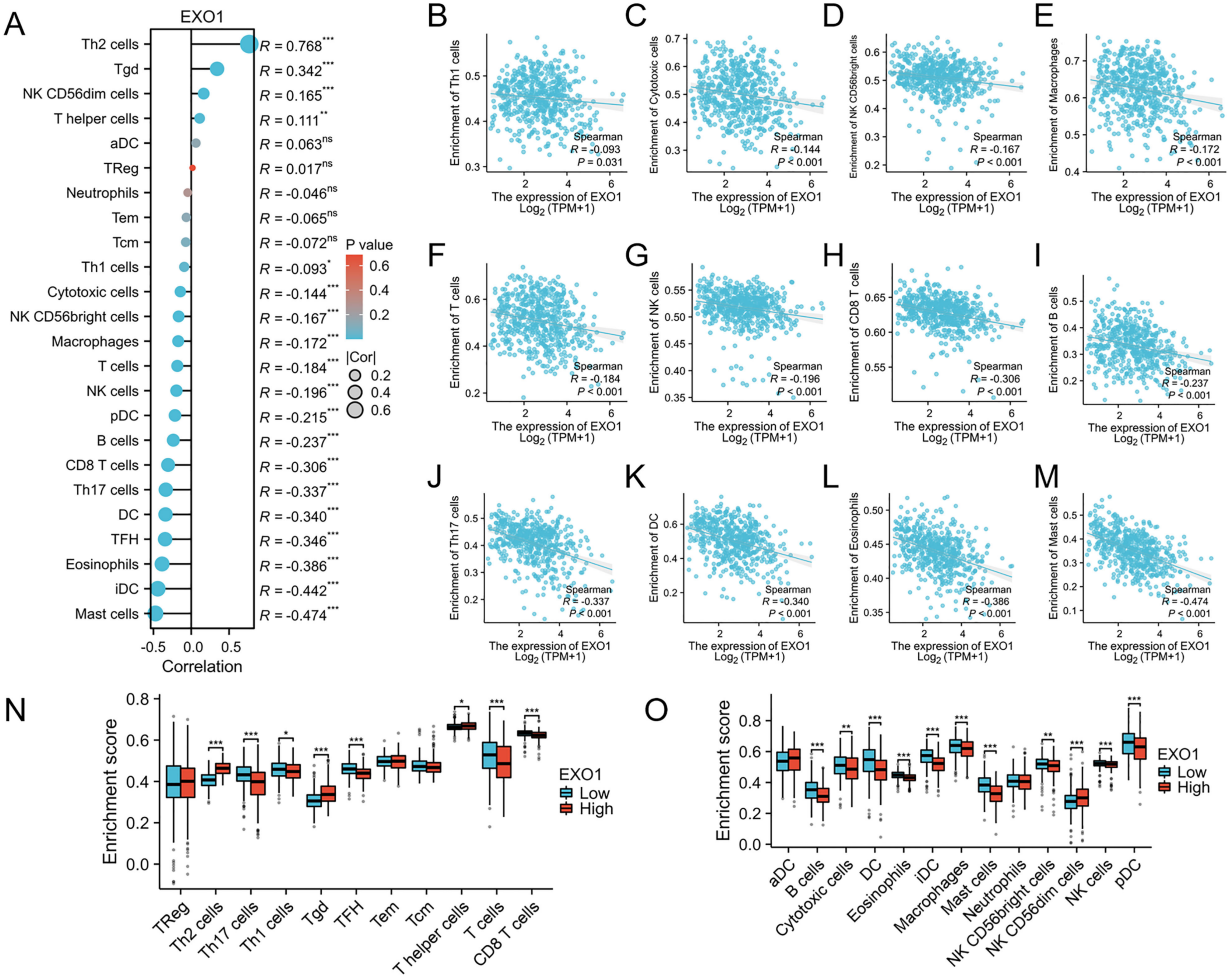
Association between EXO1 gene expression and immune cell infiltration. **(A)** The association between EXO1 gene expression and immune cell infiltration status. **(B–M)** A negative correlation exists between most immune-infiltrating cells and EXO1. **(N, O)** Variation in the enrichment of specific immune cell subsets within the high- and low-expression groups of the EXO1 gene. *p < 0.05, **p < 0.01, and ***p < 0.001.

This study further explores the relationship between EXO1 expression and immune infiltration in LUAD patients. We assessed immune-related infiltration to determine the Pearson correlation between the immune microenvironment and EXO1 expression, using ssGSEA algorithms. Our analysis revealed a positive correlation of EXO1 expression with immune infiltration levels from Th2 cells, Tgd, NK CD56dim cells, and T helper cells (**Figure 9A**). Conversely, EXO1 expression showed a negative correlation with Th1 cells, cytotoxic cells, NK CD56bright cells, macrophages, T cells, NK cells, pDC, B cells, CD8 T cells, Th17 cells, DC, TFH cells, eosinophils, iDC, and mast cells (**Figures 9A–M**). Additional analysis highlighted significant differences in EXO1 gene expression levels among various immune cell types among others (**Figures 9N, O**). This research also investigated different functional subsets of T cells, suggesting that EXO1 may play a key role in the immune-inflamed microenvironment of LUAD.

### Prognostic relevance of EXO1 expression in LUAD

**Figure 10:**
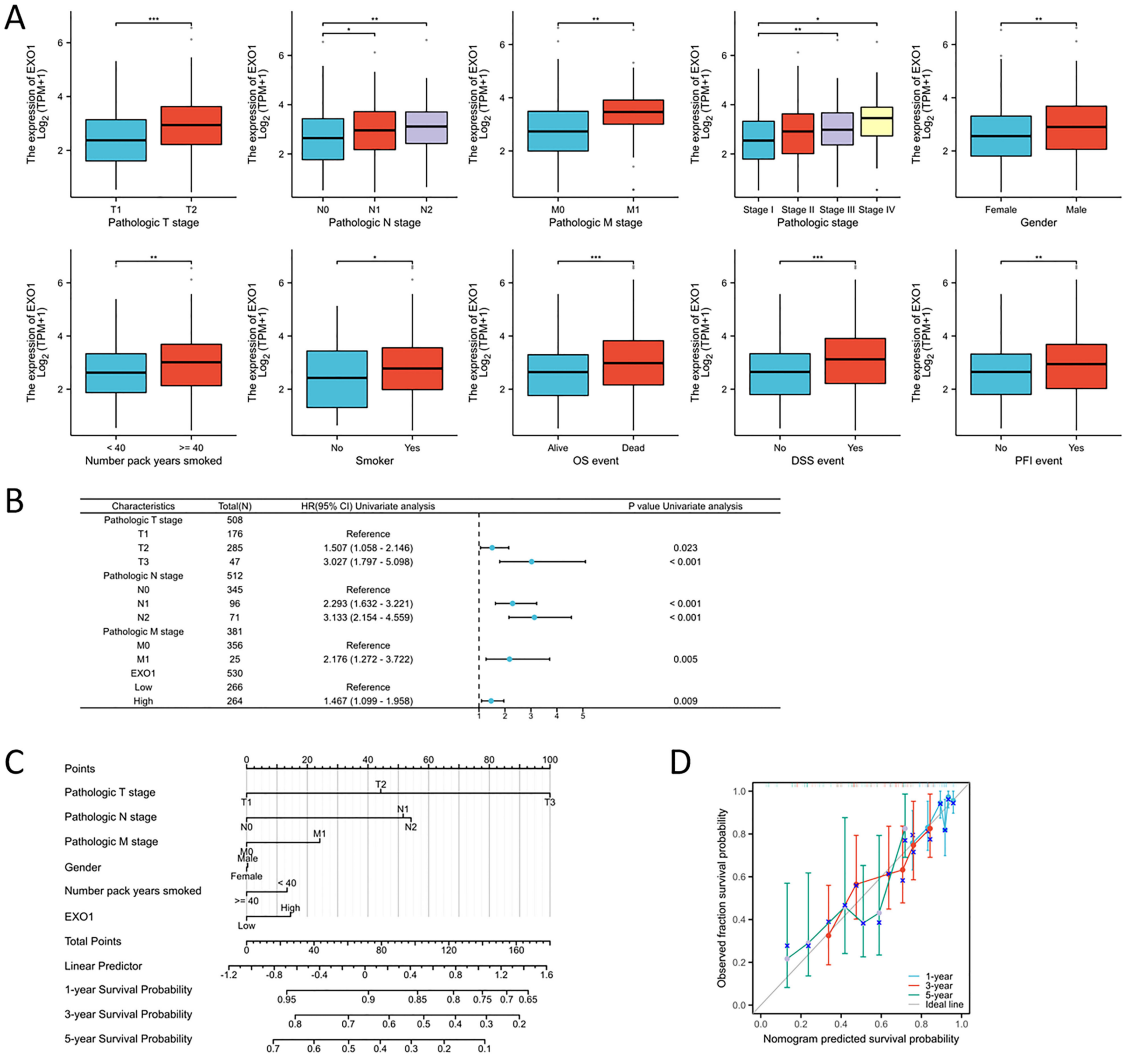
Association between EXO1 expression and clinical-pathological parameters of LUAD. **(A)** Elevated EXO1 expression levels were correlated with advanced T, N, and M stages, male gender, older age, smoking history, and an unfavorable prognosis. **(B)** Potential predictors were assessed using the univariate cox analysis. **(C)** A multivariate analysis nomogram based on clinical features associated with EXO1 expression. **(D)** The calibration chart displays the model’s prediction accuracy as determined using multi-factor Cox regression analysis. *p<0.05, **p<0.01, ***p<0.001.

The relationship between EXO1 expression and clinical parameters was evaluated using the Wilcoxon signed-rank tests. Elevated levels of EXO1 expression were associated with higher T stage, N stage, M stage, gender, and smoking status, as well as the occurrence of OS events (**Figure 10A**). Correlations between the clinicopathological characteristics of LUAD patients and their EXO1 protein levels are presented in **Table 1**. Patients with high EXO1 expression demonstrated poorer survival outcomes. These findings provide evidence supporting an association between increased EXO1 expression and advanced tumor pathological stage, thus highlighting the crucial role of EXO1 in LUAD prognosis. To advance our investigation, we employed univariate Cox regression model analysis. The subsequent findings confirmed that EXO1 characteristics could be confidently considered as reliable and independent prognostic biomarkers, significantly enhancing the accuracy of prognostic assessment for LUAD patients (**Figure 10B**). We devised a clinical prognostic risk score for LUAD, integrating T stage, N stage, M stage, clinical stage, histological grade, age, and EXO1 expression (**Figure 10C**). Furthermore, we employed a calibration chart to assess the model’s predictive accuracy (**Figure 10D**). EXO1 expression holds promise for enhancing the accuracy of predicting patients’ survival probabilities for 3- and 5-year intervals. Overall, a clear correlation between EXO1 expression and LUAD patient prognosis was evident.

**Table 1:** Association of EXO1 expression with clinicopathological characteristics in patients with LUAD.

Characteristics	Low expression of EXO1	High expression of EXO1	pvalue	Method
n	269	270		
Pathologic T stage, n (%)			0.001	Chisq test
T1	109 (20.3%)	67 (12.5%)		
T2	125 (23.3%)	167 (31.2%)		
T3	25 (4.7%)	24 (4.5%)		
T4	9 (1.7%)	10 (1.9%)		
Pathologic N stage, n (%)			0.012	Chisq test
N0	187 (35.9%)	163 (31.3%)		
N1	40 (7.7%)	57 (10.9%)		
N2	28 (5.4%)	46 (8.8%)		
Pathologic M stage, n (%)			0.013	Chisq test
M0	181 (46.4%)	184 (47.2%)		
M1	6 (1.5%)	19 (4.9%)		
Pathologic stage, n (%)			0.003	Chisq test
Stage I	166 (31.3%)	130 (24.5%)		
Stage II	55 (10.4%)	70 (13.2%)		
Stage III	35 (6.6%)	49 (9.2%)		
Stage IV	7 (1.3%)	19 (3.6%)		
Gender, n (%)			0.001	Chisq test
Female	163 (30.2%)	126 (23.4%)		
Male	106 (19.7%)	144 (26.7%)		
Number pack years smoked, n (%)			0.001	Chisq test
< 40	108 (29.3%)	80 (21.7%)		
>= 40	70 (19%)	111 (30.1%)		
OS event, n (%)			0.021	Chisq test
Alive	186 (34.5%)	161 (29.9%)		
Dead	83 (15.4%)	109 (20.2%)		
DSS event, n (%)			0.023	Chisq test
No	202 (40.2%)	181 (36%)		
Yes	49 (9.7%)	71 (14.1%)		

### Experimental validation of EXO1 protein expression in lung adenocarcinoma tissue

**Figure 11:**
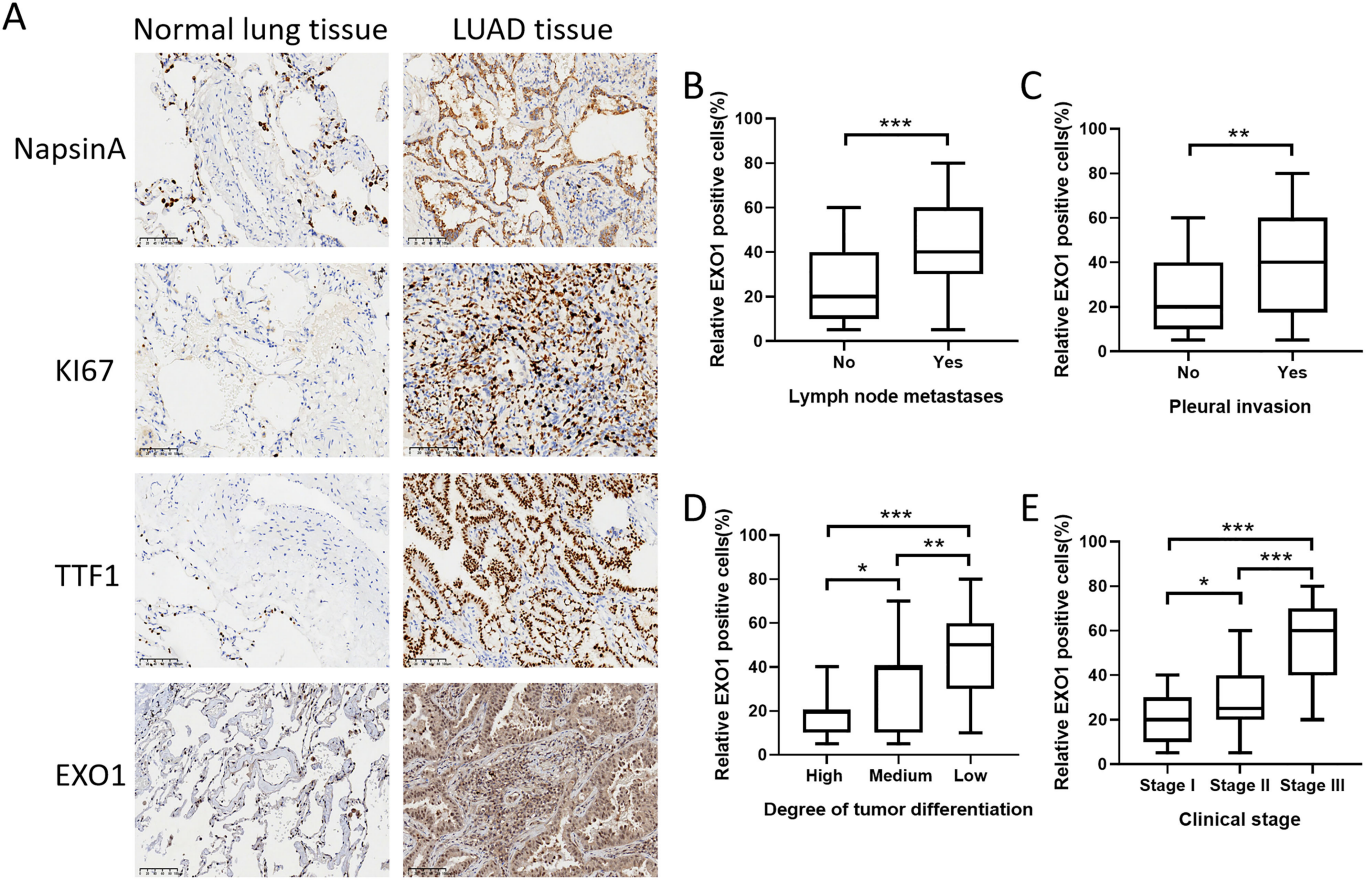
Validation of EXO1 expression in clinical samples obtained from patients with lung cancer. **(A)** The protein expression levels of EXO1, TTF1, MKI67, and NapsinA were higher in LUAD tissues compared to those in normal lung tissues. **(B-E)** The increased expression of EXO1 was correlated with lymph node metastasis, pleural invasion, high tumor differentiation grade, and advanced clinical stage. *p<0.05, **p<0.01, ***p<0.001. Patients (n) = 93.

We conducted an investigation into the variations in EXO1 protein expression between cancer-adjacent tissues and LUAD tissues by evaluating the expression levels of EXO1 and its associated proteins through immunohistochemistry (**Figure 11A**). The results revealed significantly elevated expression levels of EXO1, TTF1, MKI67, and NapsinA in LUAD tissues compared to adjacent paracancerous tissues. We evaluated the correlation between EXO1 expression and clinical parameters. Increased EXO1 expression levels were correlated with lymph node metastasis, pleural invasion, poor tumor differentiation, and advanced clinical stage (**Figures 11B–E**).

### Biological significances of EXO1 in LUAD progression

**Figure 12:**
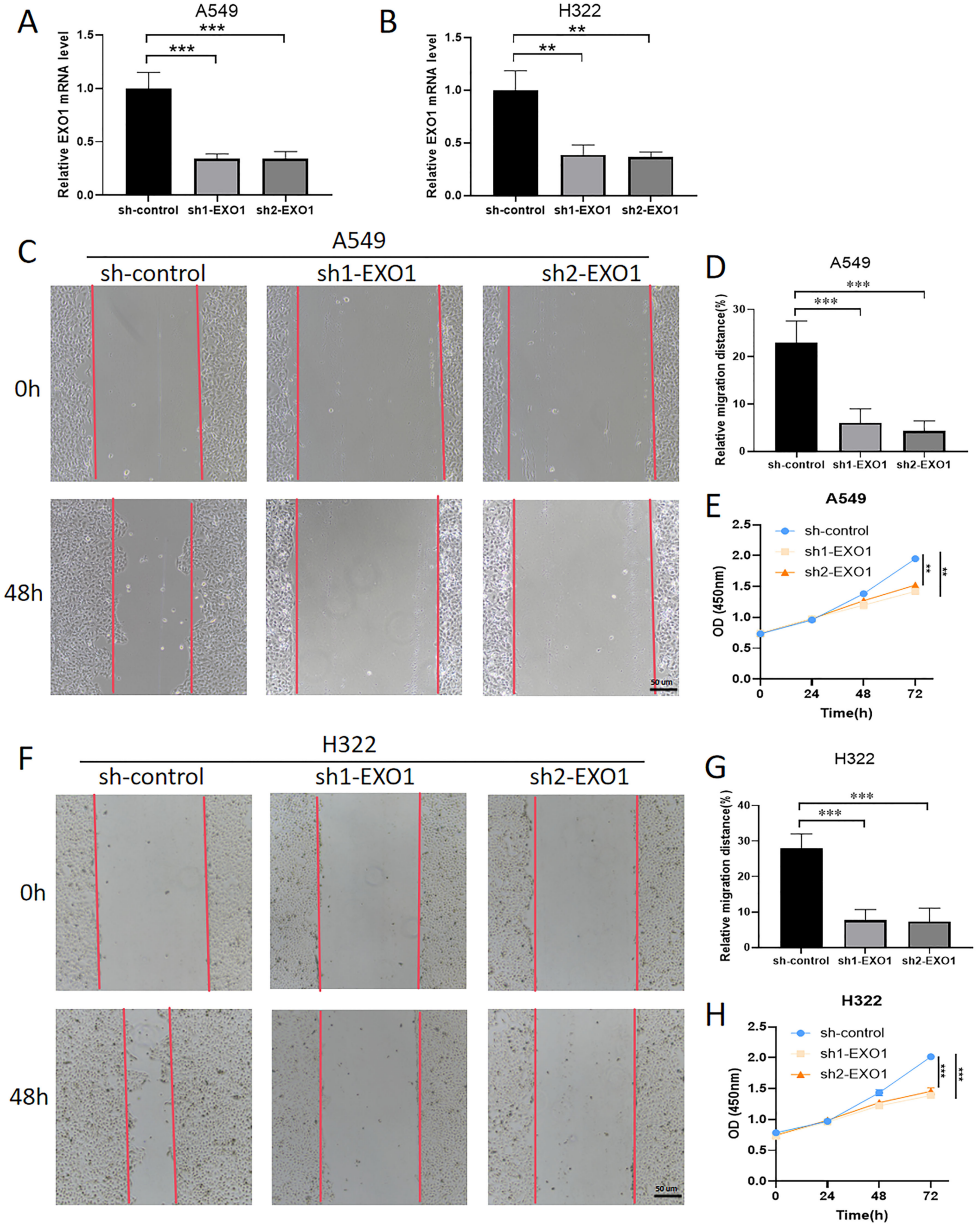
Knockdown of EXO1 inhibited LUAD progression. **(A, B)** The knockdown of EXO1 resulted in decreased mRNA levels of EXO1 in the A549 and H322 cells, as confirmed by qPCR analysis. **(C, D, F, G)** The knockdown of EXO1 inhibits the migration of A549 and H322 cells, as demonstrated by wound healing assays. **(E, H)** The knockdown of EXO1 suppresses the proliferation of A549 and H322 cells, as evidenced by CCK-8 assays. **p<0.01, ***p<0.001.

To assess the roles of EXO1 in LUAD, various *in vitro* assays were performed. As EXO1 is a promising prognostic biomarker for LUAD, we investigated its role in LUAD progression. qPCR analysis revealed that knockdown of EXO1 led to a decrease in the mRNA levels of EXO1 in A549 and H322 cells (**Figures 12A, B**). Wound healing assays showed that the metastatic ability of A549 and H322 cells was decreased after knocking down EXO1 (**Figures 12C, D, F, G**). The results of Cell Counting Kit-8 (CCK-8) showed that the proliferative ability of A549 and H322 cells was effectively weakened by knocking down EXO1 (**Figures 12E, H**). Knockdown of EXO1 inhibited the growth and migration of LUAD cells, implying that EXO1 promotes the LUAD progression.

### The inhibition of EXO1 affects the DNA damage response and the expression of DNA repair proteins

**Figure 13:**
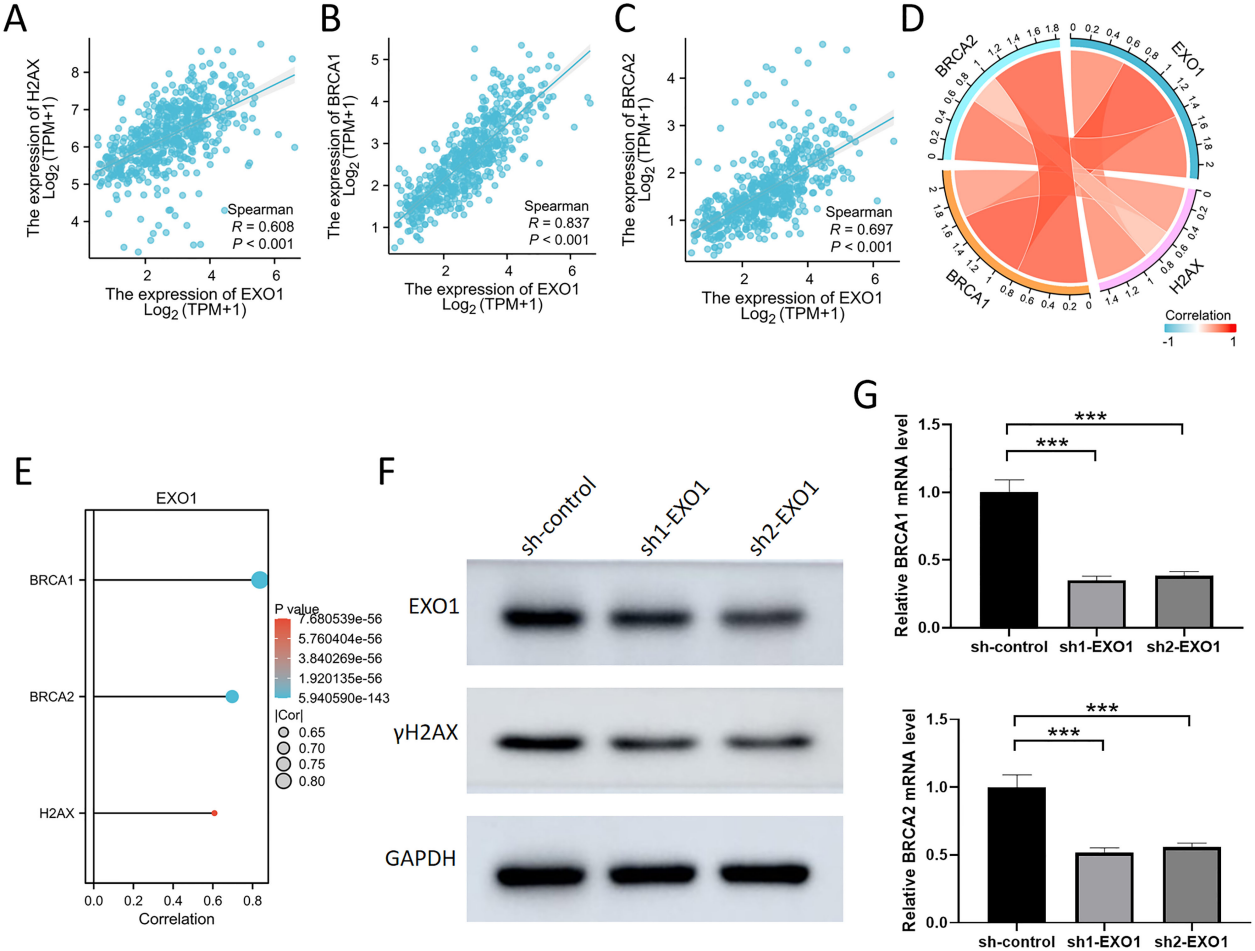
Inhibition of EXO1 reduced the expression of γH2AX, BRCA1, and BRCA2 **(A-C)** The scatter plot demonstrates a significant positive correlation between EXO1 and H2AX, BRCA1, and BRCA2. **(D)** The chord diagram illustrates a significant positive correlation among EXO1, H2AX, BRCA1, and BRCA2. **(E)** The bar graph demonstrates a significant positive correlation between EXO1 and H2AX, BRCA1, and BRCA2. **(F)** The knockdown of EXO1 resulted in decreased protein level of γH2AX in the A549 cell, as confirmed by westernblot analysis. **(G)** The knockdown of EXO1 resulted in decreased mRNA levels of BRCA1 and BRCA2 in the A549 cell, as confirmed by qPCR analysis. ***p 0.001.

To further investigate the role of EXO1 in the DNA damage response and repair processes, our results reveal a significant positive correlation between EXO1 expression and the DNA damage marker H2AX, as well as the repair proteins BRCA1 and BRCA2, in the TCGA database (**Figures 13A–E**). H2AX, an important DNA damage response protein, rapidly accumulates in its phosphorylated form (γH2AX) following DNA double-strand breaks, serving as a key marker of DNA damage. Our experiments show that knockdown of EXO1 leads to a marked decrease in γH2AX expression, suggesting that EXO1 plays a critical role in maintaining DNA damage signaling (**Figure 13F**). Furthermore, BRCA1 and BRCA2, key DNA repair proteins involved in homologous recombination, exhibited significantly reduced mRNA expression levels following EXO1 knockdown, as confirmed by qPCR and consistent with bioinformatic analyses from the TCGA database (**Figure 13G**). These findings indicate that EXO1 not only plays a crucial role in the DNA damage response but may also influence DNA repair mechanisms by regulating the expression of BRCA1 and BRCA2.

## Discussion

Utilizing Data Mining for the Exploration of Tumorigenic Mechanisms. Data mining presents itself as a robust instrument that enhances our comprehension of the intricate molecular foundations of tumorigenesis, particularly within the realm of LUAD (30–32). In our investigation, we harnessed the extensive resources offered by TCGA and GEO datasets to establish a prognostic signature specific to LUAD. A comprehensive examination of the prognostic potential of DEGs was conducted, employing both Kaplan-Meier and univariate Cox analyses, in conjunction with the LASSO Cox regression model, within the training cohort. The outcome derived was a panel of ten-gene biomarkers intricately associated with the tumor microenvironment. The architecture of the PPI networks accentuated notable interconnectivity among these ten pivotal molecules, underscored by robust connectivity scores. This collective set of genes exhibited a significant upregulation in the context of LUAD, thereby bolstering a compelling argument for their substantial implication. Subsequent survival analysis provided insights into the conspicuous correlation between elevated expression of these pivotal molecules in LUAD and the diminishment of the 5-year survival rate.

In recent years, mismatch repair gene deficiency has been proven to have a high incidence in various malignancies, such as lung cancers (33, 34). DNA damage accumulation is considered a hallmark of numerous cancers, and DNA damage repair (DDR) has emerged as a fundamental strategy for targeted cancer therapy (35). In lung cancer treatment, numerous anticancer drugs are intricately linked to DNA damage and repair systems (36). Furthermore, increased genomic mutation rates in lung cancer cells resulting from DDR abnormalities can modify the tumor immune microenvironment, thereby influencing the effectiveness of immune checkpoint inhibitors (ICIs) (37). Currently, research on the mismatch repair pathway in lung cancer is highly active (38, 39). The EXO1 protein, known for its involvement in mismatch repair and recombination, interacts with MSH2 and plays crucial roles in various diseases (40). Our immunohistochemistry experiments similarly confirmed the correlation of EXO1 and MSH2 in lung cancer. Recent studies have underscored the significant role of EXO1 in tumor immune regulation (41). However, a comprehensive understanding of its involvement in LUAD progression remains elusive. Herein, we identify EXO1 as an effective prognostic biomarker for LUAD, closely associated with immune cell infiltration within solid tumors. These findings offer novel insights into the potential role of EXO1 in LUAD, warranting further investigation.

This study further investigates genes significantly correlated with EXO1 expression in LUAD. Results reveal aberrant expression of these genes, suggesting their direct or indirect involvement in the regulatory network of EXO1, impacting the occurrence and progression of LUAD. Employing comprehensive bioinformatics methods, particularly DEG analysis, a set of 426 candidate hub genes with overlapping characteristics was identified. Through the construction of a PPI network and rigorous analytical techniques, we have developed a prognostic gene expression signature comprising ten genes: PBK, ASPM, NCAPG, EXO1, MKI67, RRM2, AURKA, DLGAP5, UBE2C, and CDC6, respectively. These genes were selected based on their established relevance to LUAD prognosis and immune infiltration. Utilizing coefficients derived from LASSO-Cox regression, we amalgamated the contributions of these ten crucial genes to establish risk score attributes. The results underscore the significance of four key genes - DLGAP5, EXO1, RRM2, and PBK - in the prediction of LUAD prognosis. Our study identified three additional candidate genes (PBK, RRM2, DLGAP5) that warrant further investigation for early diagnosis. However, the remaining six genes (ASPM, NCAPG, MKI67, AURKA, UBE2C, CDC6) were not prognostic genes, thus their potential for further research was relatively limited. Among the four genes (EXO1, PBK, RRM2, and DLGAP5), EXO1 has been extensively studied, particularly for its critical role in DNA repair and genomic stability (42). In contrast, PBK is involved in cell cycle regulation and tumorigenesis (43). While its association with cancer has been widely explored, its mechanisms are relatively complex and less directly connected to DNA repair compared to EXO1. RRM2 primarily participates in DNA synthesis and repair during cell proliferation. Although it has been investigated in certain cancers, its direct link to mismatch repair is weaker (44). DLGAP5 is associated with cell division and microtubule functions, with research largely focusing on its roles in the cell cycle and cancer proliferation rather than DNA repair processes (45). This analysis underscores EXO1’s central role in mismatch repair, establishing it as a key gene for studying DNA repair-related cancers. While PBK, RRM2, and DLGAP5 contribute to tumor biology, their roles in genomic stability and mismatch repair are less direct, making them less immediate research priorities in this context. From a therapeutic perspective, the investigation of EXO1 holds greater clinical relevance. Although PBK, RRM2, and DLGAP5 are also significant in cancer biology, EXO1’s potential impact on lung cancer mechanisms and treatment is particularly noteworthy. Consequently, focusing on EXO1 could yield deeper insights into the pathogenesis of lung cancer and identify promising therapeutic targets.

The results of the Gene Set Enrichment Analysis (GSEA) indicate that EXO1 expression is intricately linked to several pivotal molecular pathways. The Polo-like kinase 1 (PLK1) pathway is crucial for cell cycle regulation and mitotic progression, and its overexpression has been associated with poor prognosis in various cancers, including lung adenocarcinoma (LUAD) (46). The interaction between EXO1 and PLK1 may facilitate enhanced cell proliferation and survival, thereby contributing to tumor aggressiveness. Furthermore, the Forkhead box M1 (FOXM1) transcription factor is known to regulate genes involved in cell cycle progression and DNA repair, and its upregulation has been implicated in the metastatic potential of LUAD (47). Lastly, the Ataxia Telangiectasia and Rad3-related (ATR) pathway plays a critical role in the DNA damage response, and its dysregulation can lead to genomic instability, a hallmark of cancer (48). Our findings also highlight the significance of the E2F and AURORA_B pathways in relation to EXO1 expression. The E2F family of transcription factors is essential for the regulation of genes involved in cell cycle progression and apoptosis. Dysregulation of E2F activity has been linked to tumorigenesis, particularly in LUAD, where it may drive uncontrolled cell proliferation (49). The association of EXO1 with E2F suggests that it may modulate cell cycle dynamics, thereby influencing tumor growth. Similarly, the AURORA_B kinase is critical for proper chromosome segregation during mitosis, and its overexpression has been correlated with increased tumor aggressiveness and poor patient outcomes (50). Moreover, the FANCONI anemia (FA) pathway, which is involved in DNA repair and maintenance of genomic stability, has also been implicated in our analysis. The FA pathway is crucial for the repair of interstrand cross-links and is essential for maintaining cellular integrity in response to DNA damage (51). The association of EXO1 with the FA pathway suggests that it may play a role in modulating the DNA damage response in LUAD, potentially influencing tumor cell survival under genotoxic stress. Collectively, these findings underscore the multifaceted role of EXO1 in LUAD progression and its potential as a therapeutic target, warranting further investigation into its mechanistic interactions within these critical signaling pathways.

Inhibition of EXO1 activity has emerged as a promising therapeutic strategy in cancer treatment. Numerous studies have shown that specific antibodies binding to and degrading EXO1 can markedly inhibit the proliferation and survival of tumor cells (40, 52, 53). Despite previous studies indicating a significant upregulation of EXO1 in various cancers, whether its expression levels alter in lung cancer patients remains unknown. This study effectively validates the clinical significance of EXO1 in a cohort of LUAD patients by investigating its association with clinicopathological features and confirming its clinical utility. Clinical data analysis reveals a significant correlation between EXO1 expression levels and the T, N, and M stages in LUAD patients. The varying levels of EXO1 expression in tumor diagnosis and treatment highlight its potential as a biomarker. Low EXO1 expression in lung adenocarcinoma patients is associated with prolonged survival compared to those with high expression levels. Consistent with previous findings in prostate cancer, these results collectively indicate that EXO1 may act as an oncogene in the progression of malignant tumors, resulting in a worse prognosis (40). This suggests that EXO1 has the potential to serve as an independent prognostic indicator for overall survival. The use of EXO1 as a biomarker has been demonstrated to improve the accuracy and reliability of cancer detection (54, 55).

The findings of this study underscore the pivotal role of EXO1 in the progression of LUAD, particularly in relation to cell proliferation and migration. The experimental results, including wound healing assays, and CCK-8 assays, collectively demonstrate that the knockdown of EXO1 significantly impairs the proliferative and migratory capabilities of LUAD cells. In our study, the CCK-8 assay results indicate that EXO1 knockdown leads to a significant reduction in cell viability, reinforcing the notion that EXO1 is essential for LUAD cell survival and growth. This finding is consistent with previous research that has identified similar roles for other oncogenes in promoting cell proliferation (56). Additionally, the wound healing assays provide compelling evidence that EXO1 facilitates cell migration, a critical process in cancer metastasis. The mechanisms underlying this migratory capacity may involve the regulation of epithelial-mesenchymal transition (EMT), a process that has been extensively studied in the context of cancer metastasis (57). In conclusion, our study elucidates the multifaceted role of EXO1 in LUAD, particularly in relation to cell proliferation, and migration.

The limitations of this study require careful consideration. Firstly, the relatively small sample size may limit the generalizability of our findings, as a larger cohort could provide greater statistical power and enhance the reliability of the correlations observed between EXO1 expression and clinical parameters. Additionally, the lack of comprehensive *in vivo* validation restricts our ability to fully elucidate the functional implications of EXO1 in lung adenocarcinoma progression. Furthermore, the absence of long-term clinical validation raises concerns regarding the temporal stability of the identified biomarkers and their predictive capabilities in diverse clinical settings. Finally, future research should further explore the interactions between EXO1 and other proteins involved in DNA damage repair pathways. This will enhance our understanding of DNA repair mechanisms and provide potential new targets and strategies for cancer therapy. Therefore, future research should prioritize larger-scale, multicenter studies, including *in vivo* experiments such as animal model validation, as well as molecular mechanism investigations, to confirm our findings and explore the clinical applicability of the identified biomarkers in LUAD management.

## Conclusion

In conclusion, EXO1 is upregulated in advanced LUAD, which may affect the LUAD progression via key molecular functions and pathways. The acquired data suggest that EXO1 acts as a predictor of poor prognosis in LUAD and facilitates the growth and migration of lung adenocarcinoma cells.

## Data Availability

The datasets presented in this study can be found in online repositories. The names of the repository/repositories and accession number(s) can be found in the article/**Supplementary Material**.

## Supplementary material

Supplementary Table 1 The primers used in this study.

Supplementary Figure 1 Identification of DEGs between normal and LUAD groups within the dataset GSE31210.

Supplementary Figure 2 Identification of DEGs between normal and LUAD groups within the dataset GSE115002.
