# Supplementary material for: The bioinformatics analysis and experimental validation of the carcinogenic role of EXO1 in lung adenocarcinoma
Source: Front Oncol. 2024 Dec 24;14:1492725. doi: 10.3389/fonc.2024.1492725 (PMC11703735; doi:10.3389/fonc.2024.1492725)
Supplement: Supplementary Table 1 — The primers used in this study. [file Table1.docx]

| shRNA sequences | | |
| --- | --- | --- |
| EXO1 | sh1-EXO1 | GGATGTACTTTACCTTCTATT |
|  | sh2-EXO1 | GAAGTAGAGAGATCTAGAA |
| qRT-PCR primers | | |
| PBK | forward | CCAAACATTGTTGGTTATCGTGC |
|  | reverse | GGCTGGCTTTATATCGTTCTTCT |
| ASPM | forward | TGCAGTGGGTGAACATGAAAA |
|  | reverse | CGAAGAGGGTGTTACCTCGTTT |
| NCAPG | forward | AGTTCTGGCGCTTTCACGAC |
|  | reverse | GCCCGTCTAACTTCTGGATTTG |
| EXO1 | forward | CCTCGTGGCTCCCTATGAAG |
|  | reverse | AGGAGATCCGAGTCCTCTGTAA |
| MKI67 | forward | GCCTGCTCGACCCTACAGA |
|  | reverse | GCTTGTCAACTGCGGTTGC |
| RRM2 | forward | CACGGAGCCGAAAACTAAAGC |
|  | reverse | TCTGCCTTCTTATACATCTGCCA |
| AURKA | forward | GGAATATGCACCACTTGGAACA |
|  | reverse | TAAGACAGGGCATTTGCCAAT |
| DLGAP5 | forward | TAATGCCCACGTCGTTGAGAA |
|  | reverse | GCAGCTCTTGTGACTGGCTT |
| UBE2C | forward | GACCTGAGGTATAAGCTCTCGC |
|  | reverse | TTACCCTGGGTGTCCACGTT |
| CDC6 | forward | CCAGGCACAGGCTACAATCAG |
|  | reverse | AACAGGTTACGGTTTGGACATT |
| Actin | forward | AGCCTTCCTTCCTGGGCAT |
|  | reverse | CTGTGTTGGCGTACAGGTCT |
| BRCA1 | forward | TGGTTTTTATTATTTGTTTTTTAAAA |
|  | reverse | TCAACCCCAATATTTATTATTTTTC |
| BRCA2 | forward | GGTGTGGTGGTTTATGTTTGTAAT |
|  | reverse | TCAAATAATTCTCCTACCTCAACCT |
