# Supplementary figures and images for: The bioinformatics analysis and experimental validation of the carcinogenic role of EXO1 in lung adenocarcinoma
Source: Front Oncol. 2024 Dec 24;14:1492725. doi: 10.3389/fonc.2024.1492725 (PMC11703735; doi:10.3389/fonc.2024.1492725)

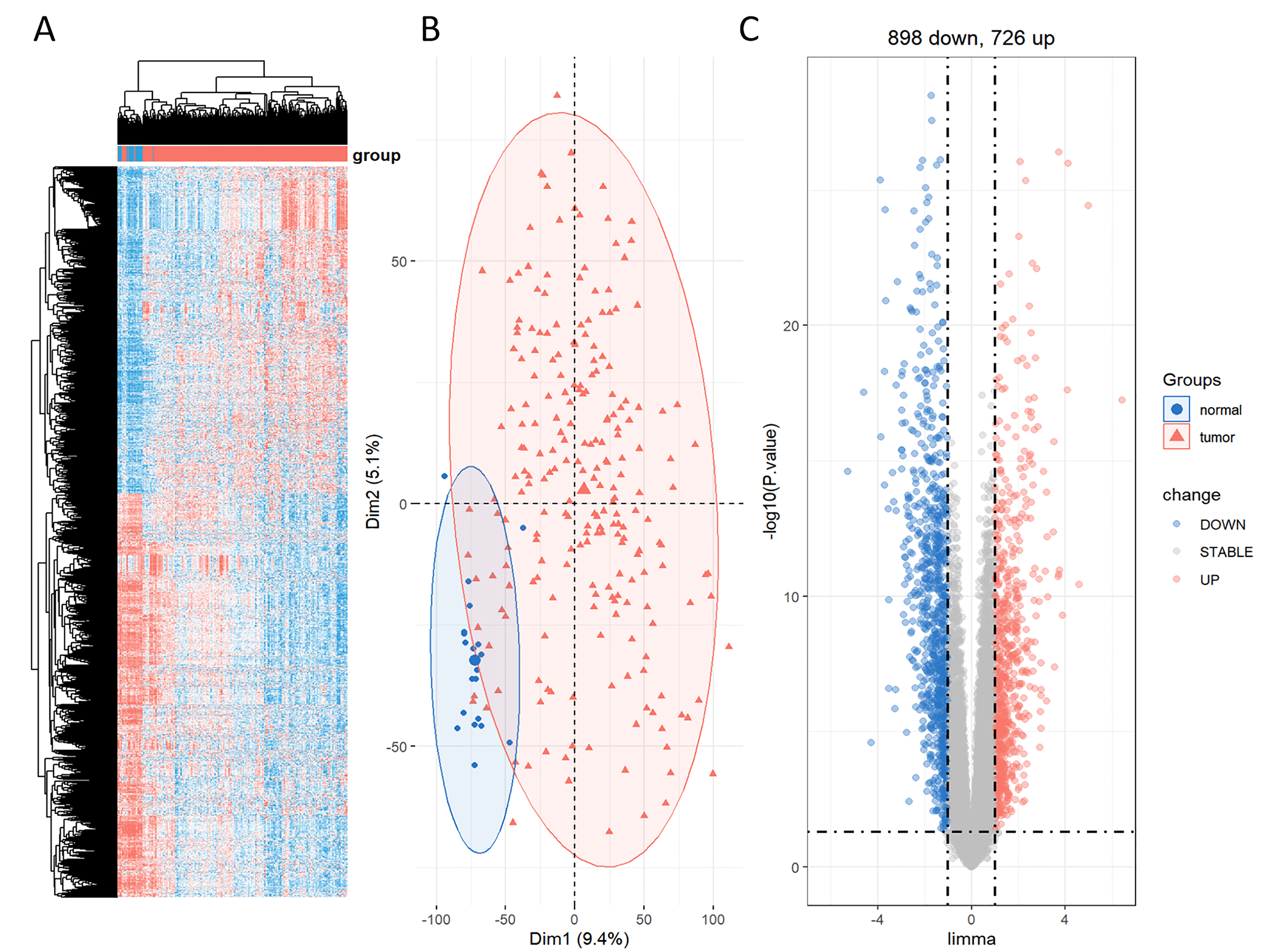

Supplement: Supplementary Figure 1 — Identification of DEGs between normal and LUAD groups within the dataset GSE31210. [file Image1.tif]

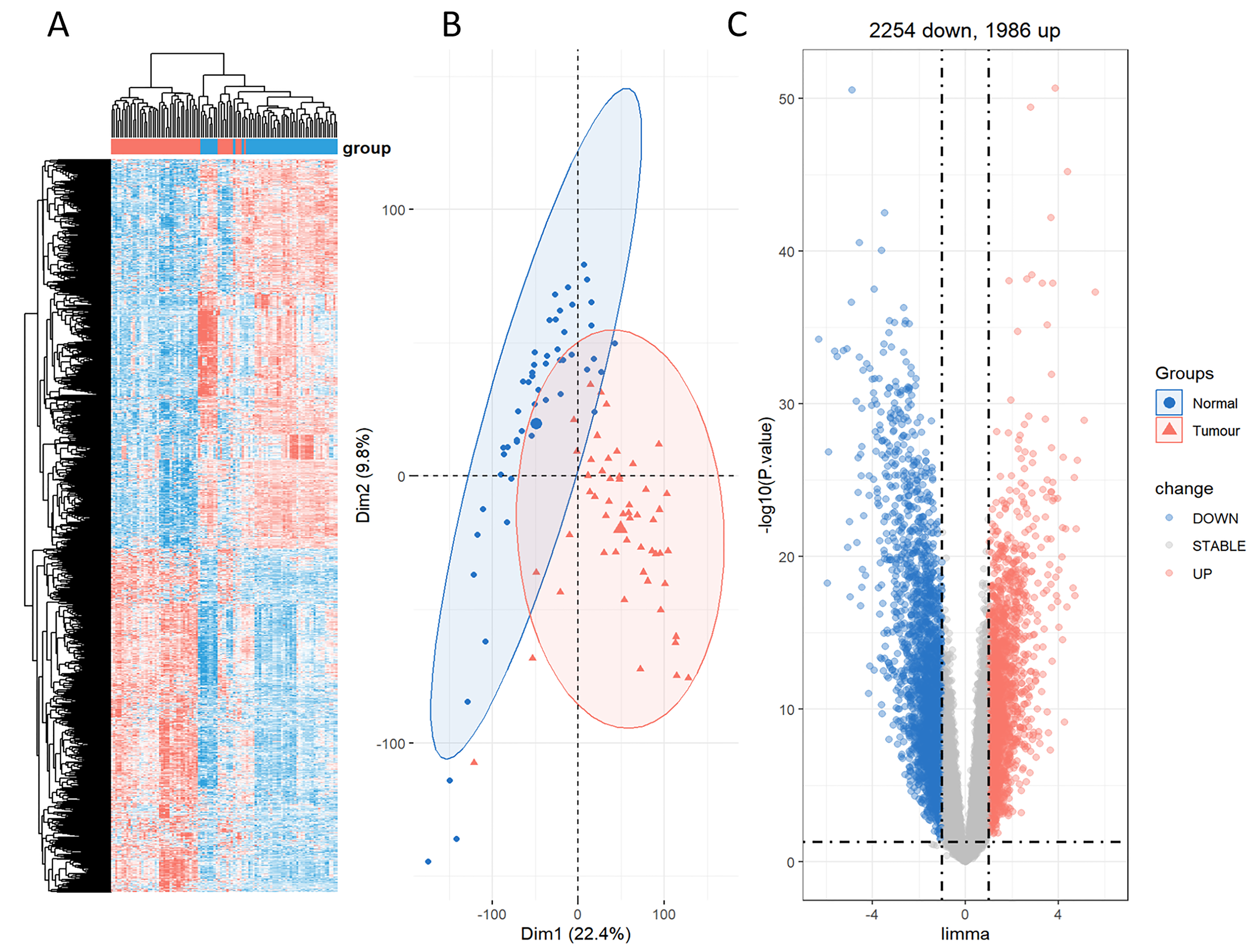

Supplement: Supplementary Figure 2 — Identification of DEGs between normal and LUAD groups within the dataset GSE115002. [file Image2.tif]
